# Facial paralysis associated with acute otitis media

**DOI:** 10.1016/S1808-8694(15)30782-5

**Published:** 2015-10-19

**Authors:** Fernando Kaoru Yonamine, Juliane Tuma, Rogério Fernandes Nunes da Silva, Maria Claudia Mattos Soares, José Ricardo Gurgel Testa

**Affiliations:** 1 Third year resident of Otorhinolaryngology and Head and Neck Surgery - Universidade Federal de São Paulo - Escola Paulista de Medicina; 2 MSc in Otorhinolaryngology; 3 MSc in Otorhinolaryngology; 4 MSc in Otorhinolaryngology. Graduate Student (PhD) in Otorhinolaryngology and Head and Neck Surgery - Universidade Federal de São Paulo - Escola Paulista de Medicina; 5 Adjunct Professor - Department of Otorhinolaryngology and Head and Neck Surgery - Universidade Federal de São Paulo - Escola Paulista de Medicina

**Keywords:** otitis media, facial paralysis

## Abstract

Acute otitis media with facial paralysis is not a very frequent association.

**Aim:**

the goal of the present investigation was to asses the evolution of facial paralysis caused by acute otitis media.

**Study format:**

clinical-retrospective.

**Materials and Methods:**

we studied 40 patients with this association, from a total of 2758 cases of facial paralysis seen during this time in the department of facial nerve disorders. All the patients were clinically assessed and had epidemiological data, prognostics and evolution.

**Results and conclusion:**

the paralysis was of sudden onset in 95% of the cases. Recovery was of 85% for grade I (House-Brackman) and 15% for grade II (House-Brackman). Treatment was clinical, with antibiotics and steroids - yielding good results. In those patients with electrical bad prognosis, facial nerve decompression turned their evolution into a favorable one.

## INTRODUCTION

Today, antibiotics have reduced the incidence of peripheral facial paralysis (PFP) associated with acute otitis media (AOM)^1^^,^^2^. They make up 1% to 4% of facial paralysis cases in many series^3^, ^4^, ^5^.

It is more common in children, with an incidence estimated in 0.004%. In adults, AOM is not so common; however this age range has up to 10 times greater chance of developing PFP as its complication^6^.

As far as treatment is concerned, the use of intravenous antibiotics, with or without steroids and myringotomy in cases of intact tympanic membranes are universally accepted. Surgery is the controversial treatment, because its effectiveness is based on personal experiences and anecdotal reports.

## OBJECTIVE

To retrospectively study cases of peripheral facial paralysis associated with acute otitis media, and to also investigate epidemiological, prognostic and development aspects.

## MATERIALS AND METHODS

This study was submitted to the Ethics in Research Committee of UNIFESP and approved under protocol # 01807/07.

We analyzed 2,758 cases of PFP seen at a tertiary hospital since 1987. From this total, 40 patients (1.45%) had PFP caused by AOM.

All patients were clinically assessed for diagnostic confirmation of acute otitis media, and they were classified according to the House-Brackmann scale during treatment onset and at its end.

The paralysis prognosis was based on electric tests with the Hilger stimulator or electroneurography.

The lesion topography was assessed though a study of tearing, stapes reflex and image exams (especially in those cases referred to surgery).

Clinical treatment was based on large spectrum antibiotics to cover the most common bacteria causing acute otitis media, associated with steroids. We also indicated eye drops and eye protection ointments.

Surgical treatment was based on myringotomy and/or mastoidectomy in order to decompress the facial nerve, without opening its sheath during the acute phase of otitis with purulent secretion.

## RESULTS

Facial paralysis associated with acute otitis media was predominantly acute and sudden in 38 (95%) patients.

Among the 40 patients, 16 were males (40%) and 24 females (60%), age varied from 4 months to 67 years, with mean age of 15 and trend of 3 years.

The initial paralysis grade was: 1 GVI (2.5%), 12 GV (30%), 15 GIV (37.5%), 11 GIII (27.5%) and 1 GII (2.5%).

Through the topographic tests we observed 90% cases of infrageniculate and only 10% of suprageniculate paralysis.

Prognosis of peripheral facial paralysis through electrophysiological tests was good in 32 (80%) patients, which were treated with antibiotics and in 4 of them myringotomy and secretion aspiration were also carried out. In 8 (20%) patients who had bad prognosis in the electrical tests, we performed mastoidectomy with facial nerve decompression in its tympanic and mastoid portions, without opening the nerve sheath. In the 8 patients submitted to nerve decompression, we observed an area of dehiscence and edema in the tympanic portion of the facial nerve.

The peripheral facial paralysis recovery was of 85% for grade I (H-B) and 15% for grade II (H-B). There were no other less favorable developments. Four (10%) already had purulent otorrhea when admitted, and in none of them there was the need for nerve decompression.

From 16 cases from which material was collected for bacteriology, we observed: 9 (56.25%) without bacterial growth, 4 (25%) haemophilus influenza, 1 (6.25%) pneumoccocus, 1 (6.25%) staphyloccocus and 1 (6.25%) streptoccocus.

All the patients assessed showed a favorable development, and in 32 (80%) patients, cure did not take longer than 3 months.

In those patients submitted to facial nerve decompression, the development was slower; however, when associated with speech and hearing therapy and physical therapy, the final development was similar to that of the non-operated group, which had better electrical prognosis.

## DISCUSSION

Of the 2,758 patients seen with PFP, 40 were due to AOM complications, in other words 1.45%. Makeham et al.^5^, in a study that analyzed the causes of PFP, found a rate of 1% caused by AOM. About 28 patients (70%) were below 12 years of age, showing a greater incidence in this age range, the age rangemost affected by AOM, as already shown in the literature^6^^,^^7^. Most of the cases (90%) had facial nerve lesion beneath the geniculate ganglion, very likely, because this is the region which is in direct contact with the infection.

Most of the middle ear secretion cultures (56.25%) were sterile, because in these cases, the patient was already under antimicrobial treatment. As far as positive cultures are concerned, the flora found was the same as that of AOM without complications, with a predominance of haemophilus influenza, very similar to what was found in the literature^6^^,^^8^^,^^9^.

In relation to the treatment of PFP caused by AOM complication, it is a consensus among most authors that the conservative approach is the one most indicated. There is a consensus among most authors that conservative treatment is the one most indicated. Intravenous antibiotic treatment against the most common AOM germs is always indicated together with steroids to treat most patients, although there is no scientific proof to use the latter^10^. Myringotomy, with or without ventilation tube insertion is also indicated in cases which the tympanic membrane is intact ^7^^,^^10^. Ellefsen et al.^6^, studying 23 children with PFP stemming from AOM, found 20 patients with intact tympanic membrane. Clinical treatment was enough for 80% of our patients, and they all developed to total improvement from the paralysis. Surgical treatment (mastoidectomy with or without facial nerve decompression) has strict indications in those patients who have a worsening of their AOM symptoms or facial paralysis even with clinical treatment and also if the patient keeps a grade VI paralysis after three weeks of treatment^6^^,^^7^^,^^10^. Patients who have a bad prognosis in electrophysiological tests also have indication of surgery^3^.

In the 8 patients submitted to mastoidectomy and facial nerve decompression we observed in the intraoperative some areas of bone dehiscence and edema of the tympanic portion of the facial nerve, and this corroborates Tschiassny's theory described by Zinis et al.^10^, in which the infectious involvement of the facial nerve happens through the bony dehiscence and neurovascular communication between the middle ear and facial nerve. Nager et al.^11^, reviewing anatomical variations of the facial nerve, found 55% Fallopian canal dehiscence; this leads us to think about other physiopathological mechanisms, since peripheral paralysis as a complication of AOM is very rare^4^^,^^6^. Another theory says that the infection causes compression to the vessels that nourish the facial nerve and this could cause local ischemia and nerve infarction, and consequent paralysis^12^. Joseph and Sperling1, analyzed the existing theories to explain its physiopathology, and concluded that the main mechanism was a direct involvement of the nerve, being by bacterial or viral toxins. It is likely that there is more than one mechanism involved in its pathophysiology.

All the patients operated evolved to PFP improvement, regardless of the initial paralysis grade.

## CONCLUSION

Assessing the results, we concluded that:

1 PFP stemming from AOM is rare;
2 Treatment with antibiotics and steroids was efficient for most patients;
3 Mastoidectomy with facial nerve decompression in cases of bad electrical prognosis caused an improvement in PFP.
